# Bipolar Disorder

**DOI:** 10.1192/pb.bp.114.050344

**Published:** 2015-12

**Authors:** Lena Jawad

*Bipolar Disorder* is a pocket-sized book whose target audience is any member of the multidisciplinary team involved in looking after a patient with bipolar disorder.

It begins with an interesting glimpse into the history of bipolar disorder and how the illness was documented in biblical texts as early as 1000 bc. Its key themes include the diagnosis of bipolar disorder, epidemiology, comorbidities, neurophysiology, genetics, psychopharmacological and non-psychopharmacological management, and managing ‘special’ populations with bipolar disorder (e.g. children, pregnant women).

The text is easy to read and key points are excellently summarised in boxes throughout. The book fulfils its aims (stated in the blurb) to a very high standard, covering all important areas of bipolar disorder in a way that is succinct and aids retention of knowledge. Although not fully comprehensive, it helpfully lists references after each chapter to guide the reader to further reading around the topic.

One of the main strengths of the book is its effective presentation of information, using a combination of prose, bullet points and diagrams to cater to the needs of a variety of learners. The chapter entitled ‘A programmatic approach to treatment’ takes readers through a thorough management plan, making the whole process more holistic – this is something I will most certainly take from the book and apply to the management of all my patients, regardless of their illness.

The only limitation is, perhaps inevitably, its emphasis on US Food and Drug Administration-approved medications in its pharmacological management chapter, which may not be as relevant to psychiatrists practising in countries other than the USA. The book, however, does its best to incorporate global knowledge of bipolar illness, with references to a variety of countries.

I would wholeheartedly recommend this guide to anyone interested in learning more about bipolar disorder or who simply requires a revision of the key topics surrounding the illness.

